# How Do Arabidopsis Seedlings Sense and React to Increasing Ambient Temperatures?

**DOI:** 10.3390/plants14020248

**Published:** 2025-01-16

**Authors:** Attila Fehér, Rasik Shiekh Bin Hamid, Zoltán Magyar

**Affiliations:** 1 Institute of Plant Biology, Biological Research Centre, H-6726 Szeged, Hungarymagyar.zoltan@brc.hu (Z.M.); 2 Department of Plant Biology, Faculty of Science and Informatics, University of Szeged, H-6726 Szeged, Hungary; 3 Doctoral School in Biology, Faculty of Science and Informatics, University of Szeged, H-6726 Szeged, Hungary

**Keywords:** ambient temperature, cell division, cell elongation, climate change, retinoblastoma-related, root, shoot, thermomorphogenesis

## Abstract

Plants respond to higher ambient temperatures by modifying their growth rate and habitus. This review aims to summarize the accumulated knowledge obtained with Arabidopsis seedlings grown at normal and elevated ambient temperatures. Thermomorphogenesis in the shoot and the root is overviewed separately, since the experiments indicate differences in key aspects of thermomorphogenesis in the two organs. This includes the variances in thermosensors and key transcription factors, as well as the predominance of cell elongation or cell division, respectively, even though auxin plays a key role in regulating this process in both organs. Recent findings also highlight the role of the root and shoot meristems in thermomorphogenesis and suggest that the cell cycle inhibitor RETINOBLASTOMA-RELATED protein may balance cell division and elongation at increased temperatures.

## 1. Introduction

Climate change is a major concern for current and future crop production efficiency and food security [1,2,3]. Not only are we expecting more frequent extreme weather events, but we are also facing the challenge of persistent increases in the average global temperature [3,4]. Temperature has a considerable impact on the development, growth, metabolism, and defense of plants [5,6] and thus largely influences the distribution of optimal crop production areas [7].

The non-stressful but significant increase in average temperatures not only accelerates the metabolism and growth of plants but also activates adaptation processes known as thermomorphogenesis [8]. Typical features of thermomorphogenesis include the elongation of hypocotyl, stem, petiole, and primary root, as well as the hyponasty of leaves with decreased surface area [5,8,9,10], which, altogether, contribute to enhanced evaporative leaf cooling [11,12,13]. Thermomorphogenesis research has initially addressed and still elaborates on two central questions: How do plants sense subtle changes in ambient temperature, and how is it translated into well-defined morphological changes?

Most of our knowledge about the molecular players involved in the response of plants to increasing ambient temperatures comes from studies of *Arabidopsis thaliana* seedlings grown at 27–29 °C (thermomorphogenesis) in comparison to 22–24 °C (typical morphogenesis). This knowledge is summarized in this review, highlighting the multitude of recently discovered molecular pathways affecting thermomorphogenesis.

## 2. Thermal Response of the Hypocotyl—A Long and Bright Story

Elongating the hypocotyl is a typical response of *Arabidopsis thaliana* seedlings to increasing ambient temperature (abbreviated as IAT from now on). Investigating its molecular background, it has become apparent that ambient light and temperature signaling share common pathways [10,14,15,16,17,18]. The photoreceptor Phytochrome B (PhyB) also functions as a thermosensor in plants [19]. PhyB alternates between a red and a far-red light-absorbing form, Pr and Pfr, respectively, with Pfr being the biologically active one. Thermal reversion of Pfr to Pr, a light-independent process, can occur in both dark and light environments and promote thermomorphogenesis. Pfr is known to translocate from the cytoplasm to the nucleus, where, together with its interacting proteins, it forms photobodies [20,21]. PhyB, due to its intrinsically disordered N-terminal extension, spontaneously undergoes liquid–liquid phase separation (LLPS) to assemble these liquid-like droplets [22]. PhyB can differentiate between light and temperature cues by undergoing conformation changes and phase separation [22]. The light-induced conformational shift from Pr to Pfr influences the assembly of PhyB condensates, whereas IAT perception directly adjusts the phase properties of PhyB droplets. Thus, specific signaling elements are integrated into the PhyB droplets in light and IAT, respectively, enabling different toggle-like regulation of PhyB signaling activity [22]. In addition to the phytochromes, the various blue light photoreceptors (the cryptochromes, the phototropins, the ZEITUPLE protein) and the UV-B sensor UVR8, also display temperature-dependent changes in their activity and thus might influence the thermomorphogenic response [23,24,25,26,27,28]. Integration of parallel light- and thermosensing in above-ground plant organs is carried out by the PHYTOCHROME INTERACTING FACTOR 4 (PIF4) transcription factor (TF) [29,30,31]. In reaction to light and thermal signals, PIF4 abundance and function is modified by both transcriptional and posttranslational mechanisms [23,27,32,33,34,35,36,37,38,39]. Furthermore, its thermomorphogenesis-promoting activity is gated by the circadian clock [32,40,41,42,43] and is confined to the epidermis in the hypocotyl [44]. PIF4 reorganizes the transcriptome in concert with various interacting proteins, including hormone-responsive TFs such as BRASSINAZOLE RESISTANT 1 (BZR1) and AUXIN RESPONSE FACTOR 6 (ARF6) [45,46]. Auxin synthesis and signaling are key targets of PIF4 during IAT-dependent hypocotyl elongation [29,47,48,49]. PIF4 directly activates the expression of YUCCA8 (YUC8), INDOLE-3-ACETIC ACID INDUCIBLE 19 (IAA19), and INDOLE-3-ACETIC ACID INDUCIBLE 29 (IAA29) coding genes. YUC8 serves as a rate-limiting enzyme in auxin biosynthesis and is essential for IAT-dependent hypocotyl elongation [47,48]. Brassinosteroids, via the TF BZR1, have also been demonstrated to act downstream of PIF4 and auxin during temperature-induced growth promotion [50,51,52]. Since BZR1 also interacts with and activates the transcription of PIF4, a feed-forward growth regulatory loop forms at IAT [50,53].

In *Arabidopsis thaliana*, in addition to those mainly relying on PhyB, there are temperature-sensing mechanisms that involve the PHYTOCHROME INTERACTING FACTOR 7 (PIF7) and the EARLY FLOWERING3 (ELF3) TFs [54,55,56,57,58,59]. The interaction between PIF7 and PIF4 results in the co-regulation of thermomorphogenesis-related gene expression, with PIF7 having the predominant effect under shade conditions [57,58,59]. PIF7 functions as both a gene expression regulator and a thermosensor [57]. At elevated temperatures, the PIF7 messenger RNA forms a hairpin within its 5′ untranslated region leading to its increased translation and the accumulation of the PIF7 protein [57]. ELF3 suppresses temperature-induced growth by repressing PIF4 expression [54,55,60]. Like PhyB, ELF3 acts as a thermosensor due to its prion-like domains, allowing temperature-dependent LLPS [60,61]. As the temperature rises, the transition in the liquid phase reduces the activity of ELF3, thus permitting PIF4 to accumulate [60,62].

Apart from the numerous potential thermosensors and the transcriptional and posttranscriptional regulators of PIF4, several epigenetic mechanisms also play a role in the complexity of thermomorphogenesis regulation [63]. These include, among others, overall H3K4me3 demethylation by JUMONJI14 (JMJ14) and JMJ15 affecting gene activation as well as repression underlying the thermomorphogenic response [64], the PIF4-mediated recruitment of the attenuator FLOWERING CONTROL LOCUS A (FCA) to the chromatin of growth-promoting genes including *YUC8* [65], histone deacetylation at the YUC8 locus promoting PIF4-binding at IAT [66,67], and PIF4-interaction with the ATP-dependent chromatin remodeling complex INO80–EIN6 ENHANCER (EEN) to activate transcription of auxin-related genes, including *YUC8* [68]. It has been observed that most of these mechanisms lead to temperature-dependent H2A.Z eviction at thermo-responsive genes to promote their transcription [66,67,68,69,70].

IAT, beside enhancing PIF4/7-mediated cell elongation, downregulates the ELONGATED HYPOCOTYL 5 (HY5) TF [31]. HY5 has long been known to control light responses, including the inhibition of hypocotyl elongation [71], among others inhibiting the expression of PIF4 target genes [72]. In the dark, the CONSTITUTIVELY PHOTOMORPHOGENIC 1 (COP1) protein translocates from the cytoplasm into the nucleus, where it promotes the degradation of HY5 to allow hypocotyl growth [72]. Warm temperatures also induce COP1 translocation and HY5 degradation [73]. HY5 would otherwise compete with PIF4 for target site binding, leading to the inhibition of thermo-responsive hypocotyl elongation [74].

**Figure 1:**
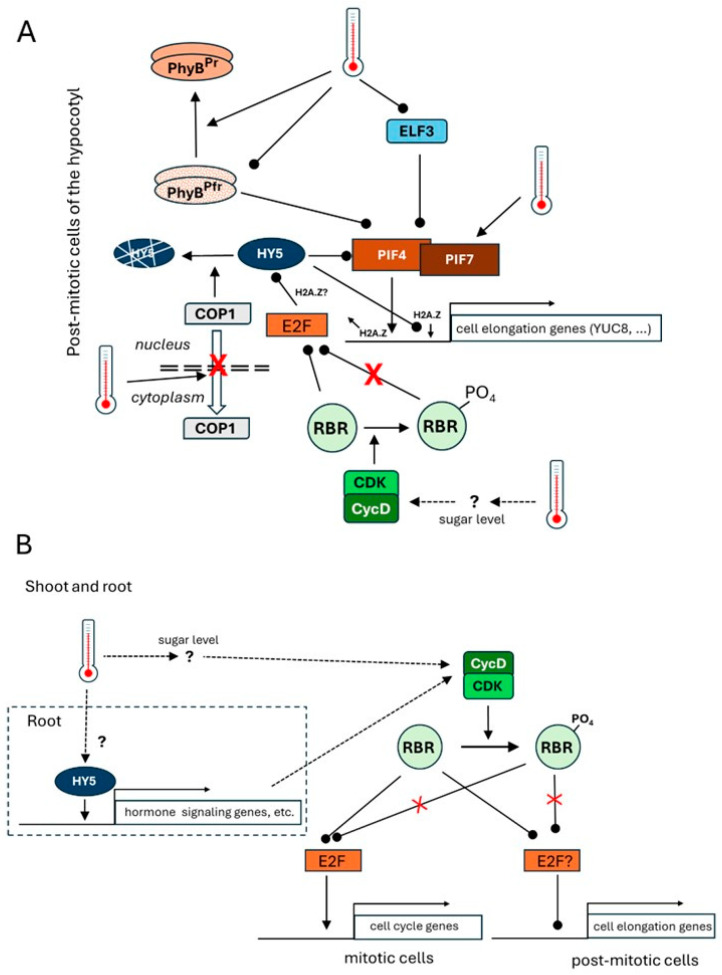
Signaling pathways involved in thermomorphogenesis of *Arabidopsis thaliana* organs. Only some of the known elements are highlighted to reduce complexity. For more detail, visit the text and the recent review by Delker et al. [83]. Dashed lines of arrows indicate hypothetical pathways. Arrows with blunt ends represent inhibition. (**A**) In post-mitotic cells of the hypocotyl, signaling from various thermosensors (PhyB, ELF3, PIF7) converges on the central PIF4 transcription regulator, directly activating the expression of cell elongation factors such as the auxin synthesis regulator YUC8. This activation includes the removal of H2A.Z histones from the promoter regions of PIF4 target genes. Increasing ambient temperatures (IATs; indicated by thermometer icons) enhance the thermal reversion of the active phytochrome PhyB^Pfr^ to PhyB^Pr^ as well as the formation of nuclear PhyB^Pfr^ droplets, thus preventing PhyB^Pfr^-mediated PIF4 degradation. The ELF3 transcription factor, a repressor of *PIF4* expression, also forms inactive droplets in response to IAT. The translation of PIF7, the co-regulator of PIF4, increases during IAT due to hairpin formation within its 5′ untranslated region. The HY5 transcription factor functions as an inhibitor of hypocotyl elongation, among others, by inhibiting *PIF4* and *YUC8* expression through the promotion of H2A.Z accumulation at their promoters. IAT results in the nuclear accumulation of COP1, which enhances HY5 degradation and consequently allows the activation of the PIF4 pathway. RBR overexpression results in IAT-dependent enhanced cell elongation in the hypocotyl. It is hypothesized that IAT increases the phosphorylation of the RBR protein in non-dividing hypocotyl cells. It might be due to the increased activity of an unknown kinase (e.g., a CycD/CDK complex) in response to the temperature-enhanced sugar metabolism and signaling. The accumulation of the phosphorylated form of RBR may inhibit *HY5* expression by an RBR and E2F-containing repressor DREAM complex (not by E2F directly, as shown for simplicity). (**B**) Both in the root and shoot meristems, IAT enhances the division of mitotic cells, likely through CycD/CDK-mediated phosphorylation and inactivation of the RBR protein. The non-phosphorylated RBR otherwise inhibits cell cycling by preventing E2F-dependent gene activation. RBR phosphorylation, however, may enhance elongation of differentiating root cells that have left the cell cycle and the meristematic region, by a similar mechanism as in the hypocotyl cells. Activation of the CycD/CDK complex in the meristems can be the consequence of IAT-enhanced sugar and/or hormone signaling. While HY5 has a negative impact on thermomorphogenesis in the hypocotyl (see part A), it is suggested to have a positive role in the IAT response of the root (boxed by dashed lines). During thermomorphogenesis of the root, HY5 directly controls several genes, many of which are related to hormone signaling and promote cell division and/or cell elongation [84]. The involvement of either HY5 or PIF4 in the IAT response of the shoot meristem is not yet known. DREAM: Dimerization partner, RB-LIKE, E2F, and MULTI-VULVAL CLASS B complex; COP1: Constitutively photomorphogenic 1; CycD: Cyclin D; CDK: Cyclin-dependent kinase; ELF3: Early flowering3; HY5: Elongated hypocotyl 5; PhyB: Phytochrome B; PIF4/7: Phytochrome interacting factor 4/7; RBR: Retinoblastoma-related; YUC8: YUCCA8.

Recently, the involvement of the *Arabidopsis thaliana* cell cycle regulatory RETINOBLASTOMA-RELATED (RBR) protein in the control of hypocotyl elongation in response to IAT has been demonstrated [75]. *Arabidopsis thaliana* RBR is also recognized as a component of the evolutionarily conserved, multi-subunits DREAM (DIMERIZATION PARTNER, RB-LIKE, E2F, and MULTI-VULVAL CLASS B) protein complex [76]. This complex primarily regulates the transition between cell division and quiescence [77,78,79]. Surprisingly, it was found that ectopic RBR expression enhanced the elongation growth of hypocotyl cells at IAT [75]. This coincided with the elevated expression of the known thermomorphogenic genes such as *PIF4*, *PIF7*, and *YUCCA8* and the downregulated expression of *HY5*. In *Caenorhabditis elegans*, the DREAM complex has been found to repress gene expression promoting gene body H2A.Z accumulation [80]. Since the DREAM complex components RBR and E2F proteins have been reported to bind the *HY5* promoter [79,81], it is tempting to speculate that this complex controls *HY5* transcription through H2A.Z deposition depending on the phosphorylation state of RBR. Enhanced RBR phosphorylation by IAT might promote the repressor activity of the complex lowering HY5 expression through H2A.Z deposition. As a result, *PIF4* expression would be released from HY5-mediated inhibition, allowing PIF4 to occupy its target sites that are otherwise blocked by HY5 [74]. HY5 and PIF4 themselves are both involved in H2A.Z-dependent modulation of gene expression. HY5 was shown to recruit members of the SWI2/SNF2-Related 1 (SWR1) histone replacement complex to its target loci, promoting gene body H2A.Z accumulation and gene repression [81], while PIF4 was shown to recruit the SWR1-related INOSITOL REQUIRING 80 (INO80) complex to remove H2A.Z from its target genes [82]. Whether the DREAM complex is involved in or interferes with these processes is a question to be investigated. A simplified summary of the known and above-discussed IAT sensing and signaling mechanisms for *Arabidopsis thaliana* hypocotyls is shown in Figure 1A. For more detail, please visit [83].

While hypocotyl elongation is widely considered the primary thermomorphogenic response of *Arabidopsis thaliana* seedlings, the activity of the shoot meristem also increases at IAT [75]. The appearance and growth of leaf primordia were faster at IAT due to both an increased size and number of their cells. In agreement with its cognate cell cycle inhibitory function, an elevated level of RBR had a negative effect on cell number but had a positive effect on cell size. Conversely, the reduced level of RBR in mutant leaves had a positive effect on cell number but negatively influenced cell size.

Based on the above, IAT might affect the balance between cell division and elongation in the dividing cells of the shoot meristem as well as in the differentiating cells in an opposite way (Figure 1B). Whether RBR might represent a hub to spatially balance morphogenetic responses to IAT is an interesting possibility to be investigated in more detail.

## 3. The Thermal Response of the Root—Hidden in the Dark

The thermomorphogenic response of the root has been less investigated compared to the hypocotyl and other above-ground plant organs [85]. The primary root may experience a rise in temperature near the soil surface; consequently, it might elongate to reach cooler soil layers that may even contain more water. The primary root growth of *Arabidopsis thaliana* can be induced by a moderate warmth of 26–29 °C in laboratory conditions, with auxin playing a key role in this process [49,85,86], although the significance of brassinosteroids has been implicated in long-term responses [87]. Thus, roots also exhibit thermomorphogenic responses, the regulation of which, however, seems to differ from that of the above-ground organs. To date, no root-born thermosensor has been identified, although research has shown that *Arabidopsis thaliana* roots can detect and respond to elevated temperatures without relying on the shoot [86,88]. Results from several laboratories illustrate that PhyB, ELF3, PIF4, and PIF7 are not directly involved in root thermomorphogenesis [84,89,90], though their action in the shoot might indirectly influence root growth [89].

Unlike the PIF TFs, the HY5 TF seems to be involved in the thermomorphogenic response of both above- and below-ground organs [84]. Albeit HY5 regulates different sets of genes in the two organs. In the shoot, it does not contribute to PIF-4-mediated hypocotyl elongation but thickness [84]. In contrast, it might play a central role in root elongation, controlling, among others, auxin and brassinosteroid signaling in PIF-4-independent ways there [84] (Figure 1B). However, its temperature-dependent effect on root growth has recently been questioned [86].

While the thermo-regulated, auxin- and brassinosteroid-dependent, elongation of the *Arabidopsis thaliana* hypocotyl primarily depends on increases in cell size and less on cell division [88], recent studies support an auxin-mediated increase in the cell division activity of the root meristem as the primary and predominant response to IAT [75,86]. However, the contribution of auxin and/or brassinosteroid-dependent cell elongation, especially under prolonged high temperature conditions, cannot be excluded in the root either [87,91]. Due to the limited number of studies conducted with different experimental setups, a comprehensive understanding of root thermomorphogenesis remains elusive. For example, reports are rather contradictory considering the IAT-dependent change in the size of the root meristem [75,86,91,92,93]. Although most studies underscore the importance of auxin in the thermo-response of the root [85], we do not yet know the link of auxin signaling either to upstream, as yet unknown, thermosensors or to downstream targets regulating cell division in the meristem. It has recently been shown, however, that IAT exerts its effect on root meristem function through the cognate, RBR-dependent, transcriptional regulation of cell division [75]. The commonly accepted model suggests that CYCLIN-DEPENDENT KINASE (CDK)-CYCLIN D complexes phosphorylate the RBR protein in response to mitogenic signals [94]. As a result, the S-phase-promoting E2F/DP TF complexes are freed from RBR-dependent inhibition, facilitating cell cycle entry. D-type cyclins with an N-terminal LXCXE RBR-binding sequence motif recruit RBR for CDK-mediated phosphorylation [95,96] serving as entry points for developmental and environmental signaling towards the cell cycle machinery [97]. The expression of CYCLIN A/D regulatory subunits of CYCLIN-DEPENDENT KINASEs (CDKs) was found to be elevated, resulting in hyper-phosphorylation of the cell cycle inhibitor RBR at a conserved CDK site, leading to its suppression at IAT. Consequently, the frequency of S-phase cells in the root meristem increased, contributing to enhanced root growth [75]. It is well accepted that D-type cyclins link the cell cycle to external signals including the availability of sugar [97,98]. Therefore, it was hypothesized that elevated ambient temperature might enhance cellular metabolism leading to sugar accumulation and cyclinD-mediated RBR phosphorylation accelerating meristem activity [75]. RBR may also be involved in controlling the elongation of differentiating cells leaving the root meristem, in a similar way to how it affects cell elongation in the hypocotyl (Figure 1). This hypothesis still needs experimental support, and the same holds true for the existence of any root-based thermosensor.

## 4. Summary and Conclusions

Increased ambient temperatures (IATs) elevate plant growth and increase the length of above- and below-ground plant organs. An increased rate of cell elongation and division at higher temperatures was long believed to be part of a general response accelerating growth without affecting morphogenesis [8]. However, extended investigations of *Arabidopsis thaliana* seedlings support the view that an intricate network of molecular players controls the adaptation of plant morphogenesis to a wide range of temperatures [8,18]. While, at the beginning of researching thermomorphogenesis, emphasis was given to cell and organ elongation in the shoot, more recently, cell division and root growth have attracted more attention. It was found that shoot and root thermomorphogenesis is controlled by diverse transcription factors (TFs), although their actions converge on auxin signaling in both organs. Furthermore, while a multitude of potential thermosensors has been described in the shoot, we do not know of any that function in the root. While cell division activity in the root meristem largely contributes to enhanced root growth at IAT, the involvement of the shoot meristem in thermomorphogenesis has hardly been investigated yet. Accumulating evidence suggests that IAT not only alters the growth rate and morphogenesis but also speeds up plant development, including developmental transitions and organ formation. During accelerated development, cell division and elongation still must be coordinated. The RBR protein can serve as a signaling hub to balance cell division and differentiation under various environmental and developmental conditions, including IAT. However, we do not know yet how it is linked to the upstream thermosensors and downstream thermomorphogenesis regulators, and how it can exert an opposite role in the meristems and in the differentiating cells in response to IAT.

The research on thermomorphogenesis in *Arabidopsis thaliana* has resulted in a wealth of molecular data, highlighting the complexity of its regulation and raising a number of questions to be answered. Further investigation of other plant species, including crops, has started to provide more insights into the process. The understanding of this process is becoming increasingly urgent in light of current climate change.
